# THE IMPACT OF COVID-19 ON RELATIONSHIPS BETWEEN CARE TEAMS AND FAMILY CAREGIVERS IN CONGREGATE CARE

**DOI:** 10.1093/geroni/igad104.1389

**Published:** 2023-12-21

**Authors:** Matthias Hoben, Emily Dymchuk, Stephanie Chamberlain, Anna Beeber

**Affiliations:** York University, Toronto, Ontario, Canada; University of Alberta, Edmonton, Alberta, Canada; University of Alberta, Edmonton, Alberta, Canada; Johns Hopkins University, Baltimore, Maryland, United States

## Abstract

Family/friend caregivers are essential in the quality of care and life of residents living in congregate care (nursing homes and assisted living). However, the COVID-19 pandemic and related public health measures substantially changed the way family/friend caregivers could be involved in resident care. It also changed the dynamic of the relationships between care teams and family/friend caregivers. While research has explored these impacts, much of the research has focused on the family/friend caregiver perspective. Our objective was to explore the impact of COVID-19 and related public health measures on these relationships from the perspective of care teams. Using videoconferencing, we conducted 9 focus groups and 2 semi-structured interviews (total of 37 participants) with managers/administrators of 6 congregate care settings and their teams (care aides, nurses, recreation and rehabilitation staff, educators). The themes we identified reflect (1) pressures care teams experienced from family/friend caregivers, (2) conflicts between care teams and family/friend caregivers, (3) support care teams received from family/friend caregivers, and (4) how care teams supported family/friend caregivers. The COVID-19 pandemic disrupted long-standing relationships between care teams and family/friend caregivers, negatively impacting care teams, family/friend caregivers, and residents. However, care teams also reported encouraging examples of successful collaboration and support from family/friend caregivers. Involving family-friend caregivers in decision making was particularly critical. Learning from these promising practices will be critical to improving preparedness for future public health crises, as well as quality of resident care and life in general.

